# Lecture on Osteo-Sarcoma, &c.

**Published:** 1839-12-07

**Authors:** Wm. Gibson


*Saturday, November 30th.*

### LECTURE ON OSTEO-SARCOMA, &c.

BY WM. GIBSON, M. D.

As I promised, I bring before you to-day, gentlemen, one of the stricture cases I showed you two weeks ago. You will recollect, at that time, we found three strictures, one an inch and a half from the mouth of the urethra, another about four inches, and the third near the bulb. With some difficulty, I then passed a bougie into his bladder; it gave him considerable pain, and produced a free discharge of blood; you will also recollect the manner in which his urine came out of the canal, spreading in several streams, and each one passing off at a right angle from the penis. I ordered an instrument to be introduced daily, and to be retained in contact with the strictures an hour or two, gradually increasing the size of the instrument. This has been done, and I now show you the improvement that has taken place: he passes his water in your presence, as before, but instead of the watering-pot-like discharge, it issues in a single stream, small, it is true, and not very rapid, but indicating a far better state of his urethra. I now introduce a rather large bougie, and you observe the ease with which it slips into the bladder, scarcely meeting any obstruction; this is the best evidence of his condition, and he may now be said to be in a very comfortable state, in which I shall leave him; continuing, however, for some time the treatment we have pursued.

On Saturday last, I showed you a man with an enormous tumour on the right side of his head, and then stated that I had seen him in the house about a year ago, but that before I could get a chance to show him to the class he had decamped, and that I had since heard nothing of him until he again presented himself this fall. You also saw, from a cicatrix through the middle of the tumour, that he had undergone an operation, and that this operation must have been merely a partial one. We found that he had been under the hands of Dr. Carpenter, a highly respectable surgeon, of Pottsville, in this state; and his own account of himself not being very satisfactory, I requested one of the residents to write to that gentleman, asking information relative to his case, &c. My own opinion was, as I stated to you, that Dr. Carpenter, judging that if the tumour was carcinomatous, and continued to grow, his patient must die, had decided to cut into it and, examining into its nature, determine whether any chance existed for its safe removal; and that, having removed a portion of it, he discovered, from its composition, the futility of any attempt to save his patient, and accordingly desisted. We have since heard from this gentleman, and judge, from the tenor of his letter, that my supposition was correct, in other words that it was a case of osteo-sarcoma. As he now stands before us, you may observe the tumour involves the anterior half of the right parietal bone, and nearly half the frontal bone on the right side, extending down to the orbitar process, and pushing the eye almost from the socket. The tumour is divided into two by the cicatrix left by the operation; this is deep enough to receive a couple of fingers, and is some three or four inches long; the surface is smooth and uniform, dense and unyielding; there are no ulcerated spots, neither any feeling of fluctuation at any one point; his sight is nearly lost from amaurosis in each eye. He traces this tumour to a kick from a horse, received on the forehead, about two years ago; from that time the tumour commenced, and has been gradually increasing; his loss of sight has also been gradual.

Osteo-sarcoma signifies the growth within a bone of a fleshy, cartilaginous or fatty mass, producing an enlargement of the original bone, often absorption, and indeed fracture of the bone, and presenting a striking analogy with cancerous affections of the soft parts; it may attack any of the bones, but has more frequently been found in those of the face, and in the ends of the long bones. Boyer, the celebrated French surgeon, found the os innominatum more often the seat of it than any bone in the body, but he is perhaps the only writer so describing it. The disease commences, ordinarily, with deep seated, lancinating pains, which exist sometimes before any swelling appears; these pains increase in violence, and already affect the constitution of the patient, before any change in the part takes place. If a limb be affected, the swelling occupies the entire circumference; it is often unequal on the surface, and studded over with knots of various dimensions, which, in advanced stages of the disease, frequently ulcerate, and discharge a thin fœtid matter; sometimes, however, the surface is plain and smooth, unyielding to pressure, and without any disposition to ulceration. Old persons are not often subject to the disease, though I have met with it in two patients beyond the age of seventy. In the worst forms of it, when the pain is excessive, the ulceration and discharge profuse, and hectic fever present, we may look for a speedy termination of the case; but we do meet with cases in which, after the excessive pain which characterises its commencement, the disease seems to be stationary, so that a patient may live a long time in a state of comfort we had no right to expect. But these are rare cases, and most generally the violence of the symptoms from the beginning predict a rapid course, and, indeed, we have examples enough to prove that a few months will finish it.

The alteration which the structure of bones undergoes in osteo-sarcoma, is very interesting. The tumour being cut into, or broken up, we get a view of numerous cells, containing a thick, cheesy, lard-like matter, or sometimes a gelatinous fluid oozes out, leaving the cells lined by a delicate membrane; the bone itself, when the disease is not far advanced, will often be found rising in numerous spicula, assuming the shape often of coral, or of certain vegetable productions, and, in some examples, instead of the cheesy or lardaceous matter, we find the softening of the bone more to resemble cartilage; the soft parts around, which have become diseased, are converted into the same matter; the muscles, ligaments, vessels and periosteum are all included in the same mass, and have undergone the same degeneration.

The causes of osteo-sarcoma are, as yet, undetermined. Syphilis, scrofula, rheumatism, and external violence have been mentioned as producing it. In the present instance, the blow upon the forehead appears to have been the exciting cause. Doubtless, the different affections I have mentioned may play a certain part in developing the disease, but instances have occurred where neither of them has existed; where, indeed, no probable cause could be suggested, and where the course has been rapid, and the termination fatal. Boyer's opinion was very clearly in favour of the action of a cancerous virus upon the bony tissue, and he was the more confirmed in it, from observing the analogy existing between the return of the disease to the stump after amputation, and the re-production of cancerous tumours after extirpation from the soft parts.

It appears sometimes as a hereditary disease, and has been transmitted successively to numerous individuals of the same family.

The prognosis of this terrible disease must always be unfavourable. The treatment of it is almost useless. Once developed, there appears to be no means of arresting its progress, and we must rely chiefly upon palliative remedies, such as are calculated to relieve pain, and quiet irritation. The last resource is the amputation of the diseased part, where it is practicable. Unfortunately, amputation itself will often prove unavailing, owing, in many instances, to constitutional disturbance. It has been ascertained, also, beyond a doubt, that the lungs and other important internal organs have been attacked in a very short time after the removal of a limb affected with the disease. In the case of this poor fellow we can do nothing; an operation has already been attempted to see whether it were possible to relieve him; it has been seen that it is not. I have no doubt at all, that both tables of the skull are diseased, as well as the dura mater, and any additional operation would be but cruelty. He is anxious himself, also, to have nothing done.

I again bring before you the little patient with hare-lip, and cleft of palate, whom you saw on Saturday last. His friends are very anxious to have his deformity removed, and I have determined to perform the operation for hare-lip; this may possibly improve the condition of his palate, also, but should it not, we can operate upon it afterwards, if it should be deemed advisable. I am rather inclined to think, however, that the case is too bad a one to afford much chance of success with the bony separation.

Neither do I promise success in the present operation, for the upper maxilla is so prominent on the right side, and the chasm between the lips so wide, that I almost fear the strain upon the two portions may be too great. The removal of the projecting portion of the right upper jaw with the teeth would probably facilitate the operation, but if it can be done without this additional pain and inconvenience, it will be far better, and I will at least give him the chance.

[Dr. Gibson now proceeded to perform the operation for hare-lip, after the manner described in the last lecture. The first cut, which was made through the edge of the right portion of the lip, was followed by the spirting of two small arteries; these were secured by ligatures. In making the cut on the opposite portion another artery sprang, and was also secured. The first pin was now passed through the two portions, at the lower edge, and the ligature passed around it; a second, in the same manner, at the upper portion of the cut; the ligatures were now pulled away, and the cut surfaces being accurately in contact no blood escaped; a third pin was now passed between the others; the points being removed, a compress was placed on either side, under the ends of the pins, and a bandage passed vertically around the head, to push forward the muscles of the cheek and lips, and a horizontal turn given to it at the lip, so as to pass over the sutures, and secure them from accidental displacement. Owing to the cries and resistance of the patient, the operation was rather more tedious and difficult than is usual.]

